# Routine Blood Count and Chemistry can predict AD dementia risk up to Ten years Ahead

**DOI:** 10.1002/alz70856_102512

**Published:** 2025-12-25

**Authors:** Amir Glik, Omry Arbiv, Keshet Prado, Orit Raphaeli, Hayim Raclaw, Roy Kait, Ofri Kait, Anat Goldstein, Chen Hajaj

**Affiliations:** ^1^ ALZAI Health Corporation, Toronto, ON, Canada; ^2^ Cognitive Neurology Clinic, Rabin Medical Center, Beilinson Hospital, Petah Tikva, Israel; ^3^ Department of Neurology, Rabin Medical Center, Beilinson Hospital, Petach Tikva, Israel; ^4^ Faculty of Medicine, Tel Aviv University, Tel Aviv, Israel; ^5^ Industrial Engineering and Management, Ariel University, Ariel, Israel; ^6^ Data Science and Artificial Intelligence Research Center, Ariel University, Ariel, Israel; ^7^ Department of Neurology, Beilinson Hospital, Rabin medical center, Petach Tikva, Israel; ^8^ Data Science and Artificial Intelligence Research Center, Ariel, Israel; ^9^ ALZAI Health Corporation, Toronto, Israel

## Abstract

**Background:**

AD dementia risk prediction may help prevent 30% of cases by treating vascular risk factors years before clinical stage. Moreover, new disease modifying drugs are given only in the earliest clinical stages. Hence, there is a need for a tool that can flag high‐risk cognitive healthy (CH) subjects in order to improve early drug accessibility. The tool should be able to screen mass populations in a short period while maintaining low cost. The aim of this study was to develop such a tool.

**Method:**

We interrogated a community cohort from the Clalit health care services. The cohort included 504,219 subjects, above the age of 45. Each subject had at least one routine blood count and basic chemistry and up to ten consecutive blood exams done on a yearly basis. The blood exams were done for various other reasons. After exclusion, there were 381,754 Cognitive Healthy (CH) subjects and 59,441 subjects diagnosed with AD (MCI or early dementia). AD Diagnosis was based on the 2011 NIA guidelines. The predictive ML tool was trained for different combinations of historical period (1 to 10y) and prediction horizons (1 to 10y). For each model, we calculated the accuracy, area under the curve (AUC), precision, recall, F1 score, false positive/negative rates, risk ratio (RR) and odds ratio (OR).

**Result:**

We present herein some examples of the model results. For one year of blood exam history (aka one blood exam) and one year of horizon prediction the accuracy was 0.73, AUC 0.8, precision 0.28, recall 0.73, F1 score 0.4. For two years of blood exam history and three years of horizon prediction the accuracy was 0.73, AUC 0.84, precision 0.28, recall 0.82, F1 score 0.42. For two years of history and ten years of horizon the accuracy was 0.73, AUC 0.81, precision 0.19, recall 0.75, F1 score 0.3.

**Conclusion:**

Routine Blood count and chemistry, done for various other reasons, may enclose information concerning future risk for AD dementia. Using ML and AI sophisticated tools can facilitate the use of routine blood exams as a screening tool for AD dementia risk assessment.

